# Supplementary material for: Cellular response upon proliferation in the presence of an active mitotic checkpoint
Source: Life Sci Alliance. 2019 May 8;2(3):e201900380. doi: 10.26508/lsa.201900380 (PMC6507650; doi:10.26508/lsa.201900380)
Supplement: Supplementary file 1 [file LSA-2019-00380_TableS1.docx]

**Table S1 – Values of normalized Stress Response Index of Figure 4C**

| **condition** | **SRI** |
| --- | --- |
| SAC-active *GAL1-MAD2* | 0.540 |
| SAC-active *tub2-401* | 0.715 |
| 21° C | -0.873 |
| YPD 6hours | 0.998 |
| Benomyl (30s) | 0.125 |
| Benomyl (10min) | 0.415 |
| Benomyl (20min) | 0.851 |
| Benomyl (40min) | 0.880 |
| *cdc23-1* | 0.969 |
| *cdc28-4* | 0.926 |
| *cdc15-2* | -0.273 |
| A2 | -0.596 |
| A3 | -0.483 |
| A9 | 0.516 |
| A10 | 0.717 |
| A13 | 0.293 |
| A14 | 0.464 |
| A15 | -0.321 |
