# Supplementary material for: Cellular response upon proliferation in the presence of an active mitotic checkpoint
Source: Life Sci Alliance. 2019 May 8;2(3):e201900380. doi: 10.26508/lsa.201900380 (PMC6507650; doi:10.26508/lsa.201900380)
Supplement: Supplementary file 2 [file LSA-2019-00380_TableS2.docx]

**Table S2 – Yeast Strains**

All strains are derivative of W303, except for yAC1700.

| **Number** | **Relevant Genotype** | **Origin** |
| --- | --- | --- |
| Ry1466 | *MATa, cdh1::HIS3, GAL-CLB2dBdel::URA3, CDC14-HA* | R. Visintin |
| yAC39 | *MATa, mad2::TRP1* | our lab |
| yAC41 | *MATa, leu2-3::LEU2::GAL1-MAD2(3x)* | our lab |
| yAC309 | *MATalpha, leu2-3::LEU2::GAL1-MAD2(3x)* | our lab |
| yAC436 | *MATa, leu2-3::LEU2::GAL1-MAD2(3x), mad3::TRP1* | our lab |
| yAC802 | *MATa, trp1::tetO_2_-CDC20-127::TRP1* | our lab |
| yAC1001 | *MATa* | our lab |
| yAC1011 | *MATalpha* | our lab |
| yAC1013 | *MATa, TUB2-mCherry::URA3* | R. Visintin |
| yAC1017 | *MATa, his3-11,15::HIS3tetR-GFP, ura3::3xURA3tetO_112_, mad2::TRP1* | S. Piatti |
| yAC1156 | *MATalpha, mad2::TRP1* | our lab |
| yAC1533 | *MATa, cdh1::HIS3* | our lab |
| yAC1700 | *MATa, CLB2-GFP::LEU2 (isogenic to S288C)* | P.A. Silver |
| yAC1708 | *MATalpha, ADE2::GALL-CDC20, cdc20::LEU2,*  *HTB2-Cherry::HIS3* | F.R. Cross |
| yAC1713 | *MATalpha, HTB2-Cherry::HIS3* | our lab |
| yAC2006 | *MATa, TUB2-mCherry::URA3, CLB2-GFP::LEU2* | our lab |
| yAC2465 | *MATa, leu2-3::LEU2::GAL1-MAD2(3x)* | our lab |
| yAC2671 | *MATa TUB2-mCherry::URA3,*  *leu2-3::LEU2::GAL1-MAD2(3x), CLB2-GFP::LEU2* | our lab |
| yAC2782 | *MATalpha, leu2-3::LEU2::GAL1-MAD2(3x)* | our lab |
| yAC2807 | *MATa, leu2-3::LEU2::GAL1-MAD2(3x),*  *trp1::tetO_2_-CDC20-127::TRP1* | our lab |
| yAC2809 | *MATalpha, trp1::tetO_2_-CDC20-127::TRP1* | our lab |
| yAC2926 | *MATa, tub2-401* | our lab |
| yAC2945 | *MATalpha, tub2-401* | our lab |
| yAC2946 | *MATa, tub2-401, mad2::TRP1* | our lab |
| yAC2970 | *MATa, CLB2-GFP::LEU2, tub2-401* | our lab |
| yAC3021 | *MATa* | our lab |
| yAC3034 | *MATa, mad2::TRP1, CLB2-GFP::LEU2, tub2-401* | our lab |
| yAC3041 | *MATa, mad2::TRP1, CLB2-GFP::LEU2* | our lab |
| yAC3076 | *MATalpha, HTB2-Cherry::HIS3* | our lab |
| yAC3078 | *MATa, HTB2-Cherry::HIS3* | our lab |
| yAC3202 | *MATa* | our lab |
| yAC3220 | *MATa, tub2-401* | our lab |
| yAC3372 | *MATa, mad2::TRP1* | our lab |
| yAC3491 | *MATa, CLB2-GFP::LEU2* | our lab |
| yAC3495 | *MATalpha, leu2-3::LEU2::GAL1-MAD2(3x),*  *CLB2-GFP::LEU2* | our lab |
| yAC3568 | *MATa* | our lab |
| yAC3582 | *MATa, leu2-3::LEU2::GAL1-MAD2(3x), cdh1::HIS3* | our lab |
| yAC3609 | *MATa, leu2-3::LEU2::GAL1-MAD2 (3x),*  *CLB2-GFP::LEU2, cdh1::HIS3* | our lab |
| yAC3650 | *MATa, (TRP1::SIC1)10x* | D.P. Toczyski |
| yAC3651 | *MATalpha, (TRP1::SIC1)10x* | our lab |
| yAC3654 | *MATa, leu2-3::LEU2::GAL1-MAD2(3x), (TRP1::SIC1)10x* | our lab |
| yAC3659 | *MATa, leu2-3::LEU2::GAL1-MAD2(3x), cdh1::HIS3, (TRP1::SIC1)10x* | our lab |
| yAC3682 | *MATalpha, cdh1::HIS3, (TRP1::SIC1)10x* | our lab |
| yAC3683 | *MATa, cdh1::HIS3, (TRP1::SIC1)10x* | our lab |
| yAC3685 | *MATa, tub2-401, (TRP1::SIC1)10x* | our lab |
| yAC3686 | *MATa, tub2-401, cdh1::HIS3* | our lab |
| yAC3694 | *MATa, tub2-401, cdh1::HIS3, (TRP1::SIC1)10x* | our lab |
| yAC3712 | *MATa, leu2-3::LEU2::GAL1-MAD2(3x)* | our lab |
| yAC3717 | *MATa* | our lab |
| yAC3883 | *MATa, leu2-3::LEU2::GAL1-MAD2(3x), CLB2-GFP::LEU2, HTB2-Cherry::HIS3* | our lab |
| yAC3885 | *MATa, leu2-3::LEU2::GAL1-MAD2(3x), CLB2-GFP::LEU2, HTB2-Cherry::HIS3, cdh1::HIS3* | our lab |
| yAC3927 | *MATa, tub2-401* | our lab |
| yAC3997 | *MATa* | our lab |
| yAC4012 | *MATa, his3-11,15::HIS3tetR-GFP, ura3::3xURA3tetO_112_, HTB2-mCherry, tub2-401* | our lab |
| yAC4013 | *MATa, his3-11,15::HIS3tetR-GFP, ura3::3xURA3tetO_112_, HTB2-mCherry* | our lab |
| yAC4018 | *MATa, his3-11,15::HIS3tetR-GFP, ura3::3xURA3tetO_112_, HTB2-mCherry, leu2-3::LEU2::GAL1-MAD2(3x)* | our lab |
| yAC4096 | *MATa, tub2-401* | our lab |
