# Supplementary material for: Cellular response upon proliferation in the presence of an active mitotic checkpoint
Source: Life Sci Alliance. 2019 May 8;2(3):e201900380. doi: 10.26508/lsa.201900380 (PMC6507650; doi:10.26508/lsa.201900380)
Supplement: Supplementary file 3 [file LSA-2019-00380_TableS3.docx]

**Table S3 – Plasmids**

| **Number** | **Description** | **Origin** |
| --- | --- | --- |
| pAC118 | *tetO_2_-CDC20-127 (TRP1)* | A. Murray (pBS94) |
| pAC149 | *tub2-401 (URA3)* | T. Huffaker (pTH18) |
